# The influence of distal colon irritation on the changes of cystometry parameters to esophagus and colon distentions

**DOI:** 10.1590/S1677-5538.IBJU.2015.0238

**Published:** 2016

**Authors:** Ezidin G. Kaddumi

**Affiliations:** 1 Department of Basic Medical Sciences, Collage of Medicine, King Saud Bin Abdulaziz University for Health Sciences, Jeddah, Saudi Arabia

**Keywords:** Urinary Bladder, Colon, Esophagus, Vagus Nerve

## Abstract

The co-occurrence of multiple pathologies in the pelvic viscera in the same patient, such as, irritable bowel syndrome and interstitial cystitis, indicates the complexity of viscero-visceral interactions and the necessity to study these interactions under multiple pathological conditions. In the present study, the effect of distal colon irritation (DCI) on the urinary bladder interaction with distal esophagus distention (DED), distal colon distention (DCD), and electrical stimulation of the abdominal branches of vagus nerve (abd-vagus) were investigated using cystometry parameters. The DCI significantly decreased the intercontraction time (ICT) by decreasing the storage time (ST); nonetheless, DED and Abd-vagus were still able to significantly decrease the ICT and ST following DCI. However, DCD had no effect on ICT following the DCI. The DCI, also, significantly decreased the Intravesical pressure amplitude (P-amplitude) by increasing the resting pressure (RP). Although DED has no effect on the P-amplitude, both in the intact and the irritated animals, the abd-vagus significantly increased the P-amplitude following DCI by increasing the maximum pressure (MP). In the contrary, 3mL DCD significantly increased the P-amplitude by increasing the MP and lost that effect following the DCI. Concerning the pressure threshold (PT), none of the stimuli had any significant changes in the intact animals. However, DCI significantly decreased the PT, also, the abd-vagus and 3mL DCD significantly decreased the PT. The results of this study indicate that chemical irritation of colon complicates the effects of mechanical irritation of esophagus and colon on urinary bladder function.

## INTRODUCTION

Due to viscero-visceral interactions, many inflammatory conditions have manifestations affecting not only the pathological organ but extends onto other viscera within the same vicinity or even further away. The spread of the symptoms onto other viscera complicates the pathological condition and make it more difficult to diagnose and manage. For example, chronic pelvic pain could be attributed to many conditions affecting urinary bladder/interstitial cystitis, gastrointestinal tract/irritable bowel syndrome, prostate gland/prostatitis or any other pelvic organ pathology (1).

Urinary bladder function can be affected by stimuli from other viscera. For example, colon inflammation in rats was shown to increase urinary bladder frequency (2). In addition, esophagus distention and electrical stimulation of vagus nerve, in rats, increased urinary bladder contraction frequency (3). In humans, patients with irritable bowel syndrome showed symptoms of urinary bladder dysfunction, such as, an increase in urination frequency, as well as an increase in residual volume (4). Moreover, patients with interstitial cystitis showed higher incidence of irritable bowel syndrome and other systemic diseases compared to controls (5).

The complexity of the viscero-visceral interactions requires the study of these interactions under different pathological conditions. Most studies were designed to show the simple viscero-visceral interaction between viscera or the direct effect of a pathological condition of one viscera on the function of another; however, it is rare to find a study on how a pathological condition of one viscera can affect the viscero-visceral interaction of other viscera. In the present study, the influence of distal colon chemical irritation on the interaction of urinary bladder with distal colon and distal esophagus distentions was investigated.

## MATERIALS AND METHODS

### Animals

Eighteen male Wistar rats (300-350g) were used in the present study. Ten of these animals had distal colon irritation and the other eight animals remained intact. Animals were purchased and housed under standard conditions in the animal house at The Hashemite University. All experimental methods were approved by the Hashemite University Institutional Board and Animal Ethical Committee, which meet the requirements of the National Institute of Health (NIH, USA) guide for the use and care of laboratory animals.

### Methods

At the day of experiment, each rat was anesthetized with urethane (1.2g/kg of 50% urethane in water); the anesthetic solution was divided into two halves, of which, one half was administered intraperitoneally and the other was given subcutaneously (6). Urethane was obtained from Sigma (St. Louis, Missouri, USA). The carotid artery and jugular vein at the left side were cannulated, in each animal, for blood pressure monitoring and anesthetic supplementation, respectively.

### Continuous cystometry

An abdominal incision was made, in each animal, in order to expose the urinary bladder and ureters. A 20-gauge needle was introduced into the dome of the urinary bladder and connected to a programmable pump (AL-1000; World Precision Instrument, Sarasota, FL). Cystometry was done by bumping normal saline at a rate of 0.25mL/min to the urinary bladder. Intravesical pressure was monitored using a pressure monitor (BP-1; World Precision Instrument, Sarasota, FL) that was connected to the needle through a pressure transducer. The temperature and hydration of the urinary bladder were preserved throughout the experiment by surrounding the urinary bladder with cotton pallets that were soaked in a warm normal saline. The ureters were tied proximal to the urinary bladder, then ureters were cut and drained distal to it.

### Distal colon distention

Distal colon distention was done by a 10mm long balloon, which was made from latex material and attached to a 25GX¾” catheter (7). The colon balloon was inserted about 10cm from the anus and was taped to the base of the tale to prevent movement. Colon balloon was connected to a 3mL syringe filled with normal saline. Distal colon distention was done manually by infusing the balloon with three increasing increments of normal saline (1mL, 2mL, and then 3mL). Last increment (3mL) produced greater-than or equal to 70mm Hg pressure, which is reported to be a noxious stimulus (8, 9).

### Distal esophagus distention

Distal esophagus distention was done by balloon, similar to the distal colon balloon. The esophagus balloon was inserted about 7cm from the upper incisors, intra-orally, to the distal esophagus (10). The balloon was connected to 1mL syringe. Esophagus distention was done by infusing the balloon with 0.5mL normal saline.

### Vagus nerve stimulation

The abdominal branches of the vagus nerve were stimulated, at the level of the esophageal opening of the diaphragm, using digital stimulator (PG 4000 A; Cygnus Technology, Inc., Delaware Water Gap, PA) and isolated using isolated current source (SIU 91; Cygnus Technology, Inc., Delaware Water Gap, PA) with isolation. The stimulation was done indirectly through the esophageal wall using a bipolar electrode that was introduced orally down to the esophagus. The stimulation was conducted at frequency of 1 train/sec with 100msec train duration. Each train was composed of 14 pulses (at 70pulses/sec). Each pulse intensity was set at 8mA with 2msec duration (11). This stimulus produced compound action potentials, which were recorded from the vagus nerve in the cervical region (12).

### Distal colon irritation

Ten rats had distal colon chemical irritation. Chemical irritation of the distal colon was done in the anesthetized animals by infusing 1mL of 2% acetic acid into the distal colon through a catheter (comprised of PE 60 tubing attached to a syringe). A cotton pallet was attached to the distal end of the catheter by fixing part of it to the distal end of the catheter tub, while leaving the remaining free part in direct contact with colon mucosa. This procedure insures the localization of the irritant within the distal colon.

## EXPERIMENT DESIGN

At the beginning of each experiment, normal cystometry recordings were performed for 30 minutes. After that, cystometry recordings were done for another 30 minutes following the infusion of the irritant (in the irritated animals). Then, cystometry recordings were done for 10 minutes with esophagus distention, followed by 10 minutes recordings without esophagus distention. In the same way, cystometry recordings for another 10 minutes were done with vagal nerve stimulation, followed by normal recordings for 10 minutes. At the end, cystometry recordings were done for 30 minutes with distal colon distentions (10 minutes for each increment of distal colon distentions; 1mL, 2mL and 3mL). Cystometry was recorded for 10 more minutes following colon balloon deflation.

All data obtained from the pressure monitor were amplified and recorded on a computer using data acquisition system (National Instruments®, www.ni.com). Cystometrograms were recorded using a BioBench software program (National Instruments®, www.ni.com). The micturition cycle timing parameters, intercontraction time (the whole micturition cycle time) (ICT); the time of the voiding phase (VT); and the time of the storage phase (ST), and the intravesical pressure parameters, resting pressure (RP); pressure threshold (PT); maximum pressure (MP); and pressure amplitude (P-amplitude; the deference between MP and RP) were measured, offline, for statistical analysis.

### Statistical analysis

All the parameters of the micturition cycles in ten minutes after each stimulus and the micturition cycles in the last ten minutes preceded the stimulus from each animal were considered for statistical analysis for each stimulus. The measurements of micturition cycles parameters preceded the stimulus were considered as control for the measurements following the stimulus. Statistical analysis was performed using two tailed, unpaired student t-test (t). Results were considered significant when P<0.05. All data are presented as mean±standard error.

## RESULTS

### Effect of Colon irritation on micturition cycle timing’s parameters

**Figure 1:**
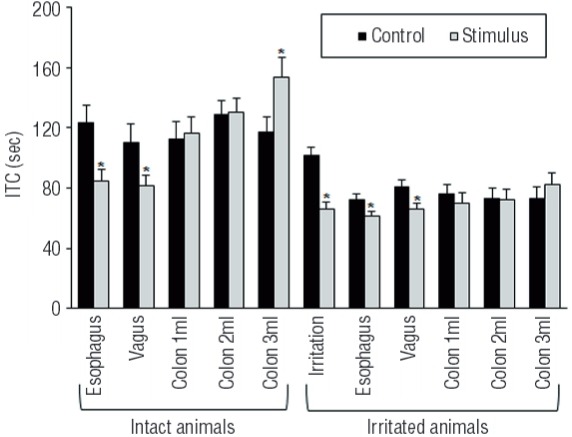
Effect of different visceral stimuli on the intercontraction time (ICT) in the intact and the irritated animals. In the intact animals, distal esophagus distention and electrical stimulation of abdominal branches of vagus nerve significantly (P ˂ 0.01 and P ˂ 0.05 respectively) decreased the ICT (data published (3)), however, distal colon distention with 3 ml significantly (P ˂ 0.04) increased the ICT (under publication). In irritated animals, chemical irritation of the distal colon significantly (P ˂ 0.001) decreased the ICT; distal esophagus distention and vagal stimulation significantly (P ˂ 0.03) decreased ICT; distal colon distention had no significant effect. Vertical bars represent standard error of the mean.

Distal colon irritation significantly increased the ICT. In addition, DCI significantly increased the ST, however, there was no significant changes to the VT following distal colon irritation. Compared to intact animals (3), in the irritated animals, distal esophagus distention and electrical stimulation of the abdominal branches of the vagus nerve were still able to significantly decrease the ICT and ST of the micturition cycles without any significant effect on the VT. On the other hand, following distal colon irritation, all three distention increments (1mL, 2mL, and 3mL) of distal colon had no significant effect on the ICT, ST, or VT, and so, all significant effects of distal colon distention on these parameters in the intact animals (under publication) disappeared following distal colon irritation. The effect of esophagus, vagal, and distal colon stimuli on the ICT are presented in Figure-1. All data for VT and ST of the micturition cycles are presented in Table-1.

**Table 1:** Effect of Different Visceral Stimuli on the Micturition Cycle Phases with and without Distal Colon Irritation.

	ST (sec)	VT (sec)
**Intact animals**		
Control	101.6±11.2	23.0±1.9
Distal Esophagus	59.9±6.3*	25.1±3.2
Control	84.2±10.6	25.8±2.3
Abd-vagus	56.8±6.1**	24.8±1.6
Control	85.0±11.1	27.4±1.8
Distal colon (1mL)	80.8±9.4	35.3±3.7
Control	98.4±9.2	30.4±1.3
Distal colon (2mL)	93.2±8.7	36.8±1.6***
Control	86.7±9.7	30.1±2.2
Distal colon (3mL)	103.4±13.0	45.0±5.0*
**Irritated animals**		
Control	80.5±4.6	21.5±1.0
Colon Irritation	46.9±3.7****	19.3±0.9
Control	52.4±3.0	20.2±0.9
Distal Esophagus	42.5±2.9*****	19.0±0.8
Control	60.0±4.5	20.7±0.9
Abd-vagus	46.8±3.4*****	19.3±0.7
Control	58.4±5.8	17.5±0.7
Distal colon (1mL)	53.1±6.2	17.2±1.2
Control	55.2±6.5	17.7±1.2
Distal colon (2mL)	53.0±6.6	19.3±1.5
Control	54.7±6.9	18.7±1.5
Distal colon (3mL)	63.4±7.4	19.0±1.7

Abd-vagus, electrical stimulation of the abdominal branches of the vagus nerve; distal esophagus, distal esophagus distention; distal colon, distal colon distention; colon irritation, chemical irritation of distal colon.

*P˂0.002, **P˂0.02, ***P˂0.004, ****P˂0.001, *****P˂0.03

### Effect of colon irritation on intravesical pressure’s parameters

In the intact animals, distal esophagus distention and electrical stimulation of the abdominal branches of vagus nerve didn’t have any significant effect on the intravesical pressure parameters (RP, PT, MP, or P-amplitude), except for a significant increase in the RP to vagal stimulation. At the same time, only 3mL distal colon distention significantly increased the MP and the P-amplitude, without any significant effect on the RP or PT. Distal colon distention with 1mL and 2mL didn’t have any significant effects on the intravesical pressure.

**Figure 2:**
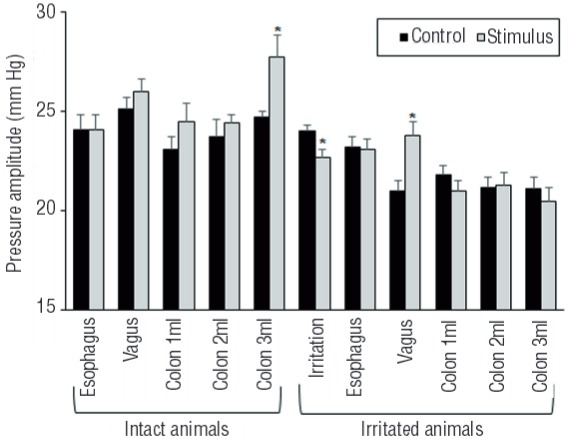
The effect of different visceral stimuli on the pressure amplitude in the intact and the irritated animals. In the intact animals, distal colon distention significantly (P ˂ 0.02) increased the pressure amplitude. In the irritated animals, distal colon irritation significantly (P ˂ 0.01) decreased the pressure amplitude, whereas, vagal stimulation significantly (P ˂ 0.003) increased the pressure amplitude. Vertical bars represent standard error of the mean.

In the irritated animals, irritation of distal colon significantly increased the RP, while significantly decreased the PT and P-amplitude. However, Irritation of distal colon had no significant effect on the MP. Following distal colon irritation, distal esophagus distention still had no significant effect on the intravesical pressure parameters. Simultaneously, distal colon distention almost had no significant effect on the intravesical pressure parameters following distal colon irritation, except for a significant increase in the PT with 3mL distal colon distention. However, in the irritated animals, electrical stimulation of the vagus nerve significantly decreased the PT, though, significantly increased the MP and P-amplitude. Vagal stimulation had no significant effect on the RP. The effect of esophagus, vagal, and distal colon stimuli on the P-amplitude are presented in Figure-2. All data for RP, PT, and MP are presented in Table-2.

**Table 2:** Effect of Different Visceral Stimuli on the Intra-vesical Pressure during Micturition Cycle with and without Distal Colon Irritation.

	RP (mm Hg)	PT (mm Hg)	MP (mm Hg)
**Intact animals**			
Control	4.6±0.2	10.8±0.8	28.7±0.6
Distal Esophagus	4.7±0.2	9.3±0.5	28.8±0.7
Control	3.8±0.3	9.1±0.5	28.9±0.6
Abd-vagus	4.7±0.2*	8.8±0.4	30.7±0.6
Control	4.1±0.3	9.8±0.6	27.1±0.6
Distal colon (1mL)	4.2±0.4	9.1±0.6	28.7±0.8
Control	3.9±0.5	9.4±0.8	27.6±0.6
Distal colon (2mL)	4.0±0.6	9.5±0.6	28.3±0.7
Control	5.6±0.3	10.7±0.6	30.3±0.4
Distal colon (3mL)	5.3±0.3	11.1±0.8	33.0±1.1**
**Irritated animals**			
Control	3.7±0.2	9.0±0.3	27.5±0.5
Colon Irritation	4.5±0.1***	7.5±0.2***	27.0±0.4
Control	4.2±0.2	7.4±0.2	27.1±0.7
Distal Esophagus	4.8±0.3	7.4±0.3	27.5±0.8
Control	4.4±0.3	8.3±0.3	25.1±0.8
Abd-vagus	4.3±0.2	6.9±0.3****	27.6±0.9**
Control	4.6±0.4	8.7±0.3	25.9±0.9
Distal colon (1mL)	4.8±0.4	8.1±0.3	25.3±0.9
Control	5.0±0.4	8.1±0.3	25.6±0.9
Distal colon (2mL)	4.7±0.4	8.4±0.3	25.5±1.0
Control	4.8±0.4	8.5±0.3	25.5±1.0
Distal colon (3mL)	4.0±0.4	7.5±0.3*	24.0±1.0

Abd-vagus, electrical stimulation of the abdominal branches of the vagus nerve; distal esophagus, distal esophagus distention; distal colon, distal colon distention; colon irritation, chemical irritation of distal colon.

*P˂0.02, **P˂0.05, ***P˂0.001, ****P˂0.005

## DISCUSSION

The results of this study shows how the changes of the urinary bladder function in response to mechanical stimuli from other viscera, either in the same region (distal colon distention) or in another region (esophagus distention), can be affected by chemical irritation to the distal colon.

The chemical irritation of the distal colon significantly increased the urinary bladder frequency. The increase in bladder frequency was attributed by the significant decrease in the storage time of the micturition cycle, where the voiding time did not have any significant changes. Since the storage phase is mainly a spinally mediated reflex (13), these results may indicate that the effect of distal colon irritation on the urinary bladder mainly occur at the spinal level. The increase of bladder frequency in response to colon irritation could be explained by neuronal sensitization. Colon irritation is proved to sensitize neurons that receive convergent inputs from colon and urinary bladder both at the dorsal root ganglion (14) and lumbosacral spinal segments (15, 16). These results are consistent with other studies, where there was an increase of bladder activity in response to colon inflammation both in rats (2) and mice (17).

The neuronal sensitization can also explain the effect of distal colon irritation on intravesical pressure, where colon irritation significantly decreased the pressure amplitude by significantly increasing the resting pressure. In addition, distal colon irritation significantly decreased the pressure threshold. The effect of colon irritation on pressure threshold could be related to sensitization of the urinary bladder afferents as well. Ustinova et al. (18) demonstrated that colon irritation in rats sensitizes urinary bladder afferents to bladder distention. Also, the decrease in pressure threshold could be related to the sensitization of the spinal neurons receiving bladder inputs. It was shown that the volume of urinary bladder distention necessary to excite the lumbosacral neurons decreased significantly following colon inflammation in rats (15). All these effects of colon irritation on the cystometry parameters reflect an increase of bladder activity.

All the same, distal colon irritation had an influence on the bladder responses to the distal colon distention and distal esophagus distention, as well as to electrical stimulation of the abdominal branches of vagus nerve. Following distal colon irritation, the distal esophagus distention and electrical stimulation of vagus nerve were still able to significantly increase the bladder frequency as it did in the intact animals. This increase in the bladder urinary frequency is still attributed to the decrease in the storage time as well (3). So, despite that colon irritation itself increased the activity of the urinary bladder, it didn’t attenuate the effect of distal esophagus and vagal stimulation on the urinary bladder activity. These results indicate that colon irritation sensitized the neural circuitries controlling the effect of esophagus and vagal stimuli to bladder activity. This sensitization is also reflected on the significant decrease of the pressure threshold by vagal stimulation in the irritated animals but not in the intact ones. The sensitization of the neural circuitries could be spinally mediated through the sensitization of the spinal part of the neural circuit that control the effect of the esophageal distention and vagal stimulation on the bladder activity, as mentioned above, or it could be directly through sensitizing the vagal afferents itself. It was shown through a neuronal tracing technique that distal colon is innervated by vagal afferents, in addition, it was shown in the same study that some nodose ganglion neurons innervate both the distal colon and urinary bladder (19).

On the other hand, following distal colon irritation, distal colon distention didn’t have any significant effect on bladder frequency, which means that distal colon distention lost its inhibitory effect on the bladder function. On the opposite, 3mL distal colon distention significantly decreased the pressure threshold following colon irritation. The ability of colon irritation to sensitize the convergent neurons for urinary bladder and colon again can explain these results. Wang et al. (20) showed that colon inflammation increased the response of the lumbosacral neurons that have long discharge “sustained neurons” following colorectal distention in the lumbosacral segments. In another study, the convergent lumbosacral neurons for colon and urinary bladder inputs showed a significant increase in their response to bladder and colon distention following colon inflammation (15). The disappearance of the inhibitory reflex of distal colon distention on the urinary bladder might be due to the sensitization of the same neuronal circuits that relays the colon irritation effects, which means that the sensitization of the colon irritation overcomes the inhibition of the colon distention or even revers it.

Finally, the setup of the present study has some limitations, mainly related to the distal colon irritation and the conscious level of the animals. More studies must be done about chronic irritation of the colon to resemble more the real pathological conditions. In addition, these experiments could be done in conscious animals, in order to exclude the effect of anesthesia on the viscero-visceral interactions. Moreover, electrophysiological studies must be carried on, in order to elaborate the changes in the neural circuitries that control the viscero-visceral interactions.

In conclusion, the results of the present study demonstrate that viscero-visceral interaction effects on the urinary bladder function depends on the variability of the mechanical and chemical stimuli from other viscera and the degree of neural overlapping that controls the different viscero-visceral interactions. The results of this study also indicate the necessity of taking in consideration the different nociceptive stimuli from other viscera in managing pathological conditions affecting certain visceral organ such as urinary bladder.
